# Hierarchically and Temporally Distinct Ca^2+^ Signaling Across Thalamic Nuclei During Nociceptive Stimulation

**DOI:** 10.1002/cns.71119

**Published:** 2026-08-31

**Authors:** Yao‐Hua Liu, Tian‐Yu Zhao, Jin Cheng, Li‐Ming Liu, Sha‐Sha Tao, Yu‐Jing Chen, Jia‐Qi Zhu, Fen‐Sheng Huang, Zhi‐Ping Cai, Fei Li, Yun‐Qing Li

**Affiliations:** ^1^ Department of Anatomy, Histology and Embryology and K. K. Leung Brain Research Centre, Basic Medical College The Fourth Military Medical University Xi'an China; ^2^ Department of Human Anatomy Baotou Medical College Baotou China; ^3^ Department of Human Anatomy, The School of Basic Medical Sciences Fujian Medical University Fuzhou China; ^4^ Department of Human Anatomy, Basic Medical College Zunyi Medical University Zunyi China; ^5^ Institute of Neuroscience and Physiology Göteborg University Göteborg Sweden

**Keywords:** Ca^2+^ signals, dynamic response pattern, fiber photometry, mouse, nociceptive stimuli, thalamus

## Abstract

**Background:**

The thalamus serves as a critical relay hub for transmitting peripheral nociceptive information to the cerebral cortex and plays a central role in the sensory, motor, affective, and cognitive dimensions of pain processing. However, the dynamic response patterns of distinct thalamic nuclei during stimulus‐pain processing remain poorly understood.

**Methods:**

In this study, fiber photometry was employed to record calcium (Ca^2+^) signals from multiple thalamic nuclei under two intensities of nociceptive somatic stimulation.

**Results:**

The results showed that all recorded nuclei exhibited rapid increases in Ca^2+^ signals following nociceptive stimulation; however, the evoked Ca^2+^ signals differed markedly in peak amplitudes and peak times across nuclei. Cluster analysis further revealed a clear spatial stratification of thalamic responses to nociceptive stimulation, with medially located nuclei generally exhibiting stronger Ca^2+^ responses than laterally located nuclei. In addition, thalamic nuclei differed in their sensitivity to stimulus intensity, with some showing pronounced intensity‐dependent Ca^2+^ responses, whereas others exhibited minimal changes. Notably, within the dorsal thalamus, the parafascicular nucleus (PF) exhibited the shortest Ca^2+^ signal peak time, whereas the posterior nucleus (PO) showed the longest. Morphological analyses further revealed distinct downstream projection targets and cortical axonal distribution patterns between PF and PO.

**Conclusions:**

This study reveals a thalamus‐wide functional stratification of neuronal Ca^2+^ dynamics during nociceptive processing, with distinct thalamic nuclei exhibiting specialized temporal profiles and intensity‐encoding properties. Collectively, these findings provide new experimental evidence supporting the view that the thalamus acts as a distributed and hierarchical hub for pain information processing.

## Graphical abstract

Fiber photometry revealed Ca^2+^ dynamics across multiple thalamic nuclei during nociceptive stimulation. Notably, all recorded thalamic nuclei were activated by nociceptive stimulation. Moreover, medial thalamic nuclei exhibited markedly stronger Ca^2+^ responses than lateral nuclei, suggesting a medial‐lateral difference in thalamic responses. In addition, distinct thalamic nuclei displayed differential sensitivity to stimulus intensity. Furthermore, *HD*‐*f*MOST analysis revealed that the parafascicular nucleus (PF) and posterior nucleus (PO) exhibited distinct temporal dynamics and projection patterns.

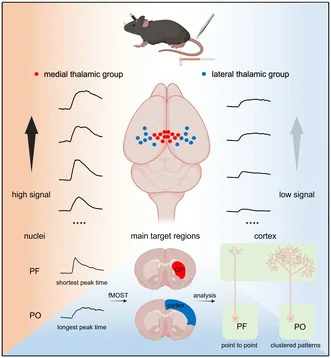

## 1 Introduction

Pain is an unpleasant sensory and emotional experience associated with, or resembling, actual or potential tissue damage [1]. It serves not only as a protective response to actual or potential injury but also a multidimensional process integrating sensory, affective, motor, and motivational components, whose central mechanisms are far more complex than early notions of pain as a simple linear pathway [2, 3, 4]. During the central transmission and regulation processes of nociceptive signals, the thalamus serves as a key relay and integrative hub, mediating the transfer and modulation of information from the spinal cord to the cerebral cortex [5, 6, 7].

Increasing evidence indicates that the thalamus is not merely a relay station for sensory information but consists of multiple functionally heterogeneous nuclei, each contributing differentially to sensory, motor, affective, and cognitive dimensions of pain [8, 9, 10]. According to the classical framework of medial and lateral pain pathways, the medial pain pathway involves thalamic nuclei including the anteromedial nucleus (AM), centromedian nucleus (CM), mediodorsal nucleus (MD), parafascicular nucleus (PF), paraventricular thalamic nucleus (PVT), and reuniens nucleus (Re) [11, 12], which project to the anterior cingulate cortex (ACC), prefrontal cortex (PFC), and insular cortex (IC), mediating higher‐order processes such as emotional, cognitive, attentional, and motivational aspects of pain [13]. In contrast, the lateral pain pathway primarily involves the posterior nucleus (PO), ventral posterolateral nucleus (VPL), and ventral posteromedial nucleus (VPM), which project to primary somatosensory cortex (S1) and secondary somatosensory cortex (S2), as well as the IC, and mediating the sensory‐discriminative aspects of pain, including localization and intensity coding [8, 14]. Beyond this functional classification, classical anatomical studies further subdivide the thalamus into distinct nuclear groups. Specifically, the anterior, medial, intralaminar, and midline nuclei were classified as the medial thalamic group, whereas the ventral, lateral, and posterior nuclei are grouped as the lateral thalamic group [15, 16]. A recent study demonstrated that consciousness‐related event potentials (ERPs) in the intralaminar and medial thalamic nuclei emerged earlier and exhibited greater amplitudes than those in the ventral thalamic nuclei [17]. Therefore, a detailed analysis of the functional specialization and dynamic response pattern among thalamic nuclei and groups during nociceptive stimulation is crucial for understanding the multidimensional central mechanisms of pain.

Previous functional magnetic resonance imaging (fMRI) studies have shown that painful stimuli activate multiple brain regions, including the thalamus [18, 19, 20]. However, due to the limited temporal resolution and spatial specificity of fMRI, it is challenging to accurately capture the dynamic activity patterns of individual thalamic nuclei during the entire stimulus‐pain process [21]. In recent years, fiber photometry has been widely applied to monitor calcium (Ca^2+^) activity in neurons of specific brain regions in freely moving animals under various conditions, including pain, emotion, arousal, sleep, aggression, and reward [22, 23, 24, 25, 26, 27]. Previous studies have reported that neuronal subpopulations across multiple thalamic regions, including VPL, VPM, PVT, and PO, are robustly activated following pain stimulation [28, 29, 30, 31, 32, 33]. Nevertheless, these studies often focus on single nuclei or isolated pathways, and the differences in stimulus paradigms, viral vectors, injection volumes, target nuclei, and mouse strains limit the ability to systematically compare Ca^2+^ signals across multiple thalamic nuclei under standardized conditions.

To address this gap, the present study employed fiber photometry to monitor Ca^2+^ signals in multiple representative thalamic nuclei under nociceptive stimuli of varying intensities, and analyzed their dynamic response characteristics and inter‐nuclear differences during nociceptive stimulation. These nuclei were selected to encompass the principal thalamic subdivisions involved in nociceptive processing, including the lateral nuclei responsible for sensory‐discriminative functions, the medial, midline, and intralaminar nuclei associated with emotional, motivational, and arousal states, and the modulatory nuclei, exemplified by the thalamic reticular nucleus (Rt). By constructing a thalamus‐wide map of pain‐related neuronal Ca^2+^ activity, this study provides new experimental evidence and theoretical support for understanding the response patterns and functional stratification of the thalamus in pain information encoding.

## 2 Materials and Methods

### 2.1 Animals

A total of 147 mice were used in this study, including 137 male C57BL/6 mice and 10 male Vglut2‐Cre mice. The C57BL/6 mice (8–12 weeks old, 20–25 g) were obtained from the Laboratory Animal Management Center of The Fourth Military Medical University (FMMU, Xi'an, China). The Vglut2‐Cre transgenic mice (8–12 weeks old, 20–25 g) were purchased from the Jackson Laboratory (Bar Harbor, ME, USA). All animals were housed under controlled temperature conditions (22°C–24°C), with free access to food and water, maintained on a 12 h light/dark cycle, and kept under stable humidity and ventilation conditions.

### 2.2 Stereotaxic Injection and Fiber Implantation

The experimental animals were divided into 16 groups (Table 1). Mice were anesthetized by injection of sodium pentobarbital (40 mg/kg i.p.) and then secured in a stereotaxic apparatus (RWD Life Science, Shenzhen, China) for surgical exposure of the skull. Injection coordinates for each thalamic nucleus were determined according to a mouse brain atlas. Viral vectors were delivered into the target nuclei of the right hemisphere using a 1‐μL microsyringe (Hamilton Company, Reno, NV, USA) at a rate of 10 nL/min. After injection, the needle was left in place for 10 min to allow for diffusion of the viral solution. A ceramic probe (RWD Life Science) was positioned 0.1 mm above the injection site and secured with dental cement. Mice were then placed on a heating pad for recovery and returned to their home cages after regaining consciousness. Detailed information on animal grouping, injection coordinates, and viral vectors is provided in Table 1.

**TABLE 1:** Grouping of experimental mice.

Group	Number and strain of mice	Injection site	Injection coordinates	Name and volume of injection reagents or viruses
1	Seven C57BL/6 mice	No	No	No
2	Ten C57BL/6 mice	AM	(AP: −0.70 mm, ML: +0.60 mm, DV: −3.85 mm)	90 μL rAAV2/9‐hSyn‐GCaMP6m‐Xc (PT‐0148, Brain VTA, Wuhan, China)
3		CM	(AP: −1.06 mm, ML: 0.00 mm, DV: −3.50 mm)	
4		LD	(AP: −1.34 mm, ML: +1.25 mm, DV: −2.90 mm)	
5		MD	(AP: −1.34 mm, ML: +0.50 mm, DV: −3.75 mm)	
6		PF	(AP: −2.30 mm, ML: +0.68 mm, DV: −3.58 mm)	
7		PO	(AP: −1.94 mm, ML: +1.25 mm, DV: −3.48 mm)	
8		PVT	(AP: −1.06 mm, ML: 0.00 mm, DV: −3.35 mm)	
9		Re	(AP: −0.58 mm, ML: 0.00 mm, DV: −4.25 mm)	
10		Rt	(AP: −0.70 mm, ML: +1.30 mm, DV: −3.95 mm)	
11		VL	(AP: −0.94 mm, ML: +1.10 mm, DV: −3.75 mm)	
12		VM	(AP: −1.46 mm, ML: +0.80 mm, DV: −4.40 mm)	
13		VPL	(AP: −1.94 mm, ML: +1.98 mm, DV: −3.98 mm)	
14		VPM	(AP: −1.94 mm, ML: +1.40 mm, DV: −3.75 mm)	
15	Five Vglut2‐Cre mice	PF	(AP: −2.30 mm, ML: +0.68 mm, DV: −3.58 mm)	150 nL CSSP‐YFP‐2E4 (BC‐SL004, BrainCase, Shenzhen, China)
16	Five Vglut2‐Cre mice	PO	(AP: −1.94 mm, ML: +1.25 mm, DV: −3.48 mm)	

### 2.3 Fiber Photometry

Three weeks after viral injection, experiments were performed using a multichannel fiber photometry system (Inper Ltd., Hangzhou, China). Prior to recording, the sampling rate was set to 60 Hz, the exposure time to 15 ms, and the camera gain to 1.0. Before each recording session, the optical fiber on the mouse's head was cleaned to remove debris and prevent potential interference with the fluorescence signal. Prior to recording, mice were connected to a patch cable (core diameter: 200 μm; numerical aperture: 0.39; RWD Life Science) and allowed to habituate in a dimly lit behavioral chamber for at least 30 min. All stimuli were applied to the left hind paw to avoid potential variability in somatosensory afferent pathways. *von* Frey filament (Stoelting, Kiel, WI, USA) stimulation of the lateral plantar surface of the left hind paw and tail pinch stimulation were applied sequentially, with an inter‐stimulus interval of at least 2 min, and a 48‐h interval between different experimental sessions. After completion of the fiber photometry experiments, all animals were perfused and sectioned to verify GCaMP6m expression and the placement of the optical fiber tips.

### 2.4 Behavioral Tests

#### 2.4.1 *von* Frey Filament Test

Prior to testing, animals were placed in Plexiglas chambers (7 cm × 7 cm × 10 cm) on a metal mesh and allowed to acclimate for at least 30 min. *von* Frey filaments of varying forces (0.6, 1.0, 1.4, and 2.0 g) were applied perpendicularly to the lateral plantar surface of the left hind paw. A positive response was defined as an obvious paw withdrawal, flinching, or licking in response to the filament [34]. Each stimulus intensity was applied five times, and the paw withdrawal frequency (PWF) was calculated as the number of positive responses divided by the total number of stimuli. In the nociceptive behavioral scoring assay, the plantar surface of the hind paw was stimulated six times per animal.

#### 2.4.2 Tail Pinch Test

Animals were acclimated in the same apparatus as described above. During the experiment, the base of the mouse tail was pinched using metal forceps, and nociceptive behavioral responses as well as Ca^2+^ signal changes were recorded. In the nociceptive behavioral scoring assay, the tail of each animal was stimulated six times.

### 2.5 Brain Tissue Preparation

After deep anesthesia was induced by injection of sodium pentobarbital (80 mg/kg i.p.), mice were transcardially perfused with 50 mL of 0.01 mol/L phosphate‐buffered saline (PBS, pH 7.4), followed by slow perfusion with 200 mL of 0.1 mol/L phosphate buffer (PB, pH 7.4) containing 4% paraformaldehyde. After perfusion, brains were removed and post‐fixed in the same fixative for 4–6 h, and then transferred to a 30% sucrose solution prepared in PB until they sank. Coronal brain sections (30 μm thick) were cut using a cryostat (CM1950, Leica Microsystems GmbH, Wetzlar, Germany) and collected sequentially into three sets in 6‐well plates containing PBS, followed by three washes before further processing.

### 2.6 Histological Verification

The first set of brain sections was examined under a fluorescence microscope (BX‐60, Olympus Corporation, Tokyo, Japan) to verify viral expression and the accuracy of optical fiber placement. After confirmation, the second set of brain sections was rinsed three times and incubated with DAPI diluted in PBS (1:1000; sc‐3598, Thermo Fisher Scientific, USA) for 5 min, followed by three additional washes, and then mounted with fluorescent mounting medium and coverslipped. All brain sections were imaged using a whole‐slide scanning system (VS200, Olympus Corporation) and a laser confocal microscope (FV1000, Olympus Corporation). Images were exported in TIFF format using FV31S‐SW software (Olympus Corporation). To further verify the accuracy of viral expression and optical fiber implantation sites, the second set of sections containing the fiber tracks was removed from the slides for Nissl staining, followed by imaging using the whole‐slide scanning system. The third set of brain slices was stored in cryoprotectant solution.

### 2.7 *HD* ‐ *f*MOST

On Day 28 after viral injection, mice in Groups 15 and 16 were perfused, and the brains were harvested and embedded in resin. The samples were subsequently immersed in a propidium iodide (PI) solution and subjected to automated serial sectioning and imaging using the High‐resolution fluorescence micro‐optical sectioning tomography system (*HD*‐*f*MOST, BioMapping 1000, Woyi Technology, Wuhan, China), with a spatial resolution of 0.32 × 0.32 × 1.0 μm^3^. The acquired *f*MOST dataset included a YFP channel for axonal tracing and a PI channel for image registration [35]. The PI channel images were registered to the Allen CCFv3 standard mouse brain atlas to obtain spatial transformation matrices, which were then used to reconstruct YFP‐labeled nerve fibers in three dimensions.

### 2.8 Calcium Signal Analysis

After histological verification, mice with inaccurate injection sites, misplaced optical fibers, or poor signal quality were excluded. Ultimately, no fewer than seven animals were included in each experimental group for further analysis (Figure S2). Data from mice with confirmed viral expression and correct optical fiber placement were subsequently imported into Inper Data Process software (Inper Ltd., Hangzhou, China) for analysis. Changes in fluorescence intensity were quantified as Δ*F*/*F* = (*F – F*_0_)/*F*_0_, where *F* represents the fluorescence intensity at each time point and *F*_0_ denotes the baseline fluorescence intensity. A temporal marker was set at the onset of each event, and fluorescence signals within a time window spanning 2 s before and 6 s after the event were extracted for analysis. The processed data were subsequently imported into Prism 9 (GraphPad Software, San Diego, CA, USA) for visualization and quantitative analysis, including line plots, heatmaps, and calculation of the area under the curve (AUC) to assess neuronal Ca^2+^ signals. Peak amplitude was defined as the maximum Ca^2+^ signal reached following stimulation, whereas peak time referred to the time required to reach this maximum value. The slope was calculated as the ratio of the peak Ca^2+^ amplitude to the corresponding peak time. Hierarchical clustering analysis was performed using the Ward.D2 method implemented in RStudio (Posit Software, Boston, MA, USA).

### 2.9 Data Statistics and Analysis

All experimental data are presented as mean ± standard error of the mean (SEM). Differences in AUC between two groups were analyzed using an unpaired *t*‐test. Comparisons of nociceptive behavioral scores induced by the two types of stimuli, as well as comparisons of responses to different stimulus intensities within the same thalamic nucleus, were performed using paired *t*‐tests. The thresholds for statistical significance were set at *p < 0.05, **p < 0.01, ***p < 0.001, and ****p < 0.0001.

## 3 Results

### 3.1 Nociceptive‐Related Behavioral Responses and Ca^2+^ Signal Changes Induced by Different Stimulus Intensities

**FIGURE 1:**
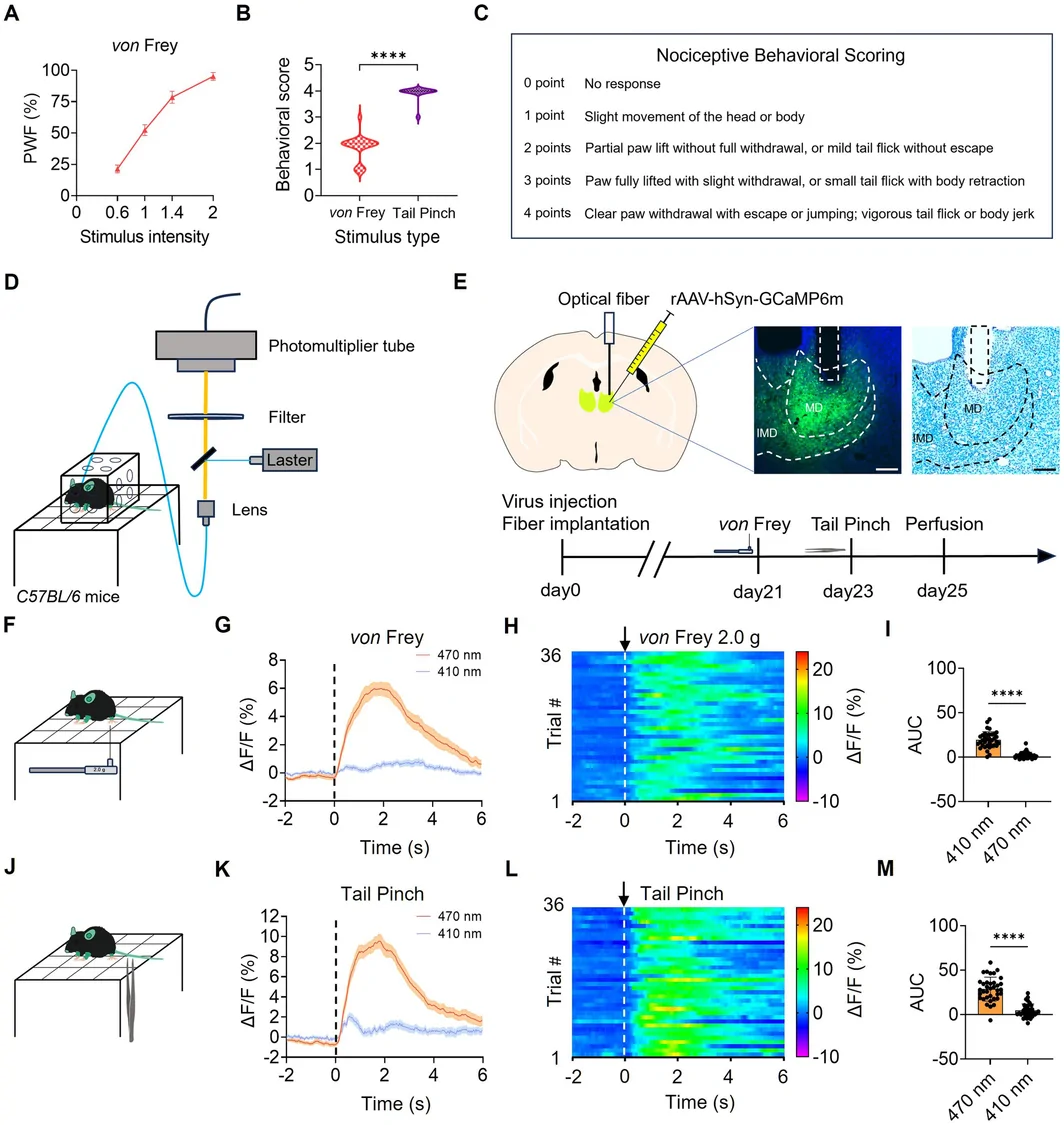
Nociceptive behavioral responses and fiber photometry recordings in the MD under different stimulus intensities. (A) Percentage of paw withdrawal responses evoked by *von* Frey filaments of four different forces. (B) Nociceptive behavioral scores induced by *von* Frey filament and tail pinch stimulation (Paired *t*‐test, *n* = 7, ****p < 0.0001). (C) Scoring criteria for nociceptive behaviors (Score 0: No response; 1: Slight movement of the head or body; 2: Slight paw lift without full withdrawal or mild tail movement; 3: Complete paw withdrawal or tail movement accompanied by mild body twisting; 4: Vigorous paw withdrawal with escape or jumping behavior, or pronounced tail flicking and body contraction). (D) Schematic illustration of the fiber photometry recording system. (E) Schematic diagram of viral injection strategy, representative injection sites, Nissl staining of the injection location, and the experimental timeline. Scale bar = 200 μm. (F) Schematic illustration of the *von* Frey stimulation paradigm. (G) Representative Ca^2+^ signal traces in the MD evoked by *von* Frey stimulation. (H) Heat map showing trial‐by‐trial Ca^2+^ signal changes in the MD in response to *von* Frey stimulation. (I) AUC of Ca^2+^ signals in the MD evoked by *von* Frey stimulation (Unpaired *t*‐test, *n* = 9, ****p < 0.0001). (J) Schematic illustration of the tail pinch stimulation paradigm. (K–M) Representative Ca^2+^ signal traces, heat maps, and corresponding AUC of MD responses evoked by tail pinch stimulation (*n* = 9, Unpaired *t*‐test, ****p < 0.0001).

To characterize thalamic Ca^2+^ responses evoked by noxious somatic stimulation, we first established an appropriate and reliable stimulation paradigm. Four graded *von* Frey filaments (0.6, 1.0, 1.4, and 2.0 g) were sequentially applied to the lateral plantar surface of the left hind paw in Group 1 mice, and paw withdrawal frequency (PWF) was recorded. The results showed that PWF increased progressively with stimulus intensity. Specifically, a 1.0 g stimulus elicited a PWF of 52%, whereas a 2.0 g stimulus induced a PWF of up to 96% (Figure 1A). To ensure consistent evocation of withdrawal responses and minimize variability in Ca^2+^ signals caused by motor differences, a 2.0 g *von* Frey filament was selected as a lower‐intensity nociceptive stimulation paradigm for subsequent experiments.

Tail pinch, a commonly used high intensity noxious stimulus, robustly elicits pain‐related behavioral responses. Previous studies have demonstrated that tail pinch induces stronger Ca^2+^ responses than *von* Frey or brush stimulation, indicating its effectiveness in evoking pain‐related neural activity [36]. Based on these findings, tail pinch was adopted as a high intensity nociceptive stimulus in the present study. According to previously established nociceptive behavioral scoring systems [37, 38, 39], the immediate responses of mice to noxious stimuli were classified into five grades ranging from 0 to 4. Subsequently, both *von* Frey and tail pinch stimuli were sequentially applied to mice in Group 1. The results showed that tail pinch elicited significantly higher nociceptive behavior scores than the milder *von* Frey stimulation (Figure 1B,C). These findings indicate that the 2.0 g *von* Frey stimulus and tail pinch reliably evoke nociceptive behavioral responses, providing two nociceptive stimulation paradigms with different intensities for subsequent thalamic Ca^2+^ signal analyses.

Finally, MD was selected as an example to demonstrate the fiber photometry recording procedure. A recombinant adeno‐associated virus encoding the genetically encoded calcium indicator GCaMP6m was injected into the right MD, followed by optical fiber implantation. Fiber photometry recordings revealed that both types of noxious stimuli elicited robust increases in Ca^2+^ signals within the MD. Histological analysis confirmed accurate viral expression and fiber placement within the MD, consistent with anatomical boundaries (Figure 1D–M). These results suggest that the MD plays an important role in the perception and transmission of nociceptive information.

### 3.2 Ca^2+^ Signal Responses in Different Thalamic Nuclei Evoked by *von* Frey Filament and Tail Pinch Stimulation

**FIGURE 2:**
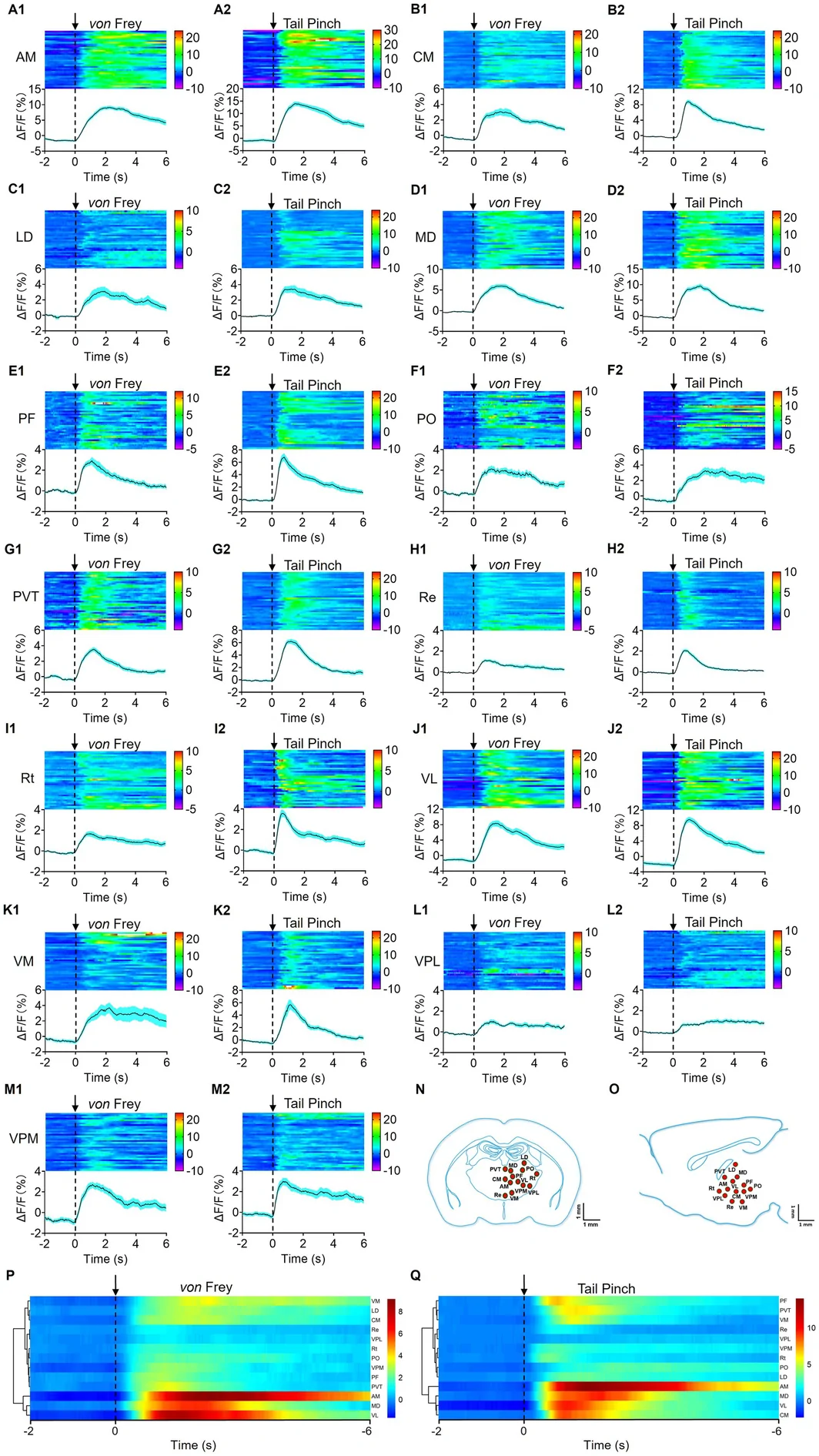
Ca^2+^ signal responses in thalamic nuclei evoked by *von* Frey filament and tail pinch stimulation. (A–M) Representative Ca^2+^ signal traces and corresponding heatmaps from 13 thalamic nuclei in response to *von* Frey filament and tail pinch stimulation. (N) Coronal anatomical schematic illustrating the locations of the 13 thalamic nuclei. (O) Sagittal anatomical schematic illustrating the locations of the 13 thalamic nuclei. (P) Hierarchical clustering analysis of the averaged Ca^2+^ responses of 13 thalamic nuclei evoked by *von* Frey stimulation. (Q) Hierarchical clustering analysis of the averaged Ca^2+^ responses of 13 thalamic nuclei evoked by tail pinch stimulation. Scale bar = 1 mm.

To examine the Ca^2+^ dynamics of thalamic neurons in response to noxious somatic stimulation, recombinant adeno‐associated virus encoding the genetically encoded calcium indicator GCaMP6m (rAAV‐hSyn‐GCaMP6m) was injected into the thalamic nuclei of mice in Groups 2–14, including CM, MD, PF, PO, PVT, Re, VPL, VPM, AM, Rt, lateral dorsal nucleus (LD), ventral lateral nucleus (VL), and ventral medial nucleus (VM). Morphological assessment confirmed robust viral expression in all targeted nuclei, with the fiber tips located within the virus expressing regions. Nissl staining further verified that fiber placement corresponded accurately to the anatomical boundaries of each thalamic nucleus (Figure S1). Subsequently, Ca^2+^ responses to *von* Frey filament and tail pinch stimulation were recorded sequentially from all 13 nuclei. All nuclei exhibited increased Ca^2+^ signals in response to the nociceptive stimuli, and heatmap analyses demonstrated high reproducibility and stable induction across repeated trials. Notably, the amplitude and temporal profiles of Ca^2+^ signals varied across nuclei (Figure 2A–O), indicating potential functional heterogeneity among thalamic nuclei in response to nociceptive stimulation. To further examine inter‐nuclear similarities and differences in Ca^2+^ responses to noxious stimulation, hierarchical clustering analysis was performed based on the mean Ca^2+^ signals evoked by *von* Frey filament stimulation for each nucleus (Figure 2P,Q). Using the Ward.D2 method [40] to compute the distance matrix, clustering analysis revealed four distinct groups corresponding to different Ca^2+^ signal intensities: Cluster 1 (ultra‐high signal) included only AM; Cluster 2 (high signal) included MD and VL; Cluster 3 (medium signal) included VM, LD, and CM; and Cluster 4 (low signal) included Re, VPL, Rt, PO, VPM, PF, and PVT. Under tail pinch stimulation, Cluster 1 (ultra‐high signal) again included only AM; Cluster 2 (high signal) included MD, VL, and CM; Cluster 3 (medium signal) included PF, PVT, and VM; and Cluster 4 (low signal) included Re, VPL, VPM, Rt, PO, and LD.

In summary, all thalamic nuclei exhibited significant increases in Ca^2+^ signals in response to both mild and strong nociceptive stimuli, suggesting that these nuclei may participate in the encoding, integration, and ascending transmission of nociceptive information. Notably, hierarchical clustering consistently classified AM into the ultra‐high signal group, MD and VL into the high signal group, and Re, VPL, VPM, Rt, and PO into the low signal group under both stimulation paradigms. These findings suggest that the thalamus may process nociceptive information through a functionally stratified organization.

### 3.3 Hierarchical Clustering Analysis of All Trials Across Thalamic Nuclei Under Two Types of Noxious Stimuli

From the clustering analysis of mean Ca^2+^ signals under the two different pain intensities, we observed a potential medio‐lateral anatomical distribution of thalamic responses to noxious stimuli. Medially located nuclei generally exhibited higher Ca^2+^ signals, whereas laterally positioned nuclei displayed lower responses, with this difference being particularly distinct under the high‐intensity tail pinch stimulation. To further validate this observation, all trials (*n* = 468) from 13 thalamic nuclei under both types of noxious stimuli were pooled, and hierarchical clustering was performed using the Ward.D2 method to compute the distance matrix. The proportion of trials for each nucleus within a cluster was then used to determine the cluster assignment of each nucleus under different stimulus intensities. The results revealed that, under both stimulation conditions, thalamic nuclei could be grouped into four clusters corresponding to distinct levels of Ca^2+^ signal intensity.

**FIGURE 3:**
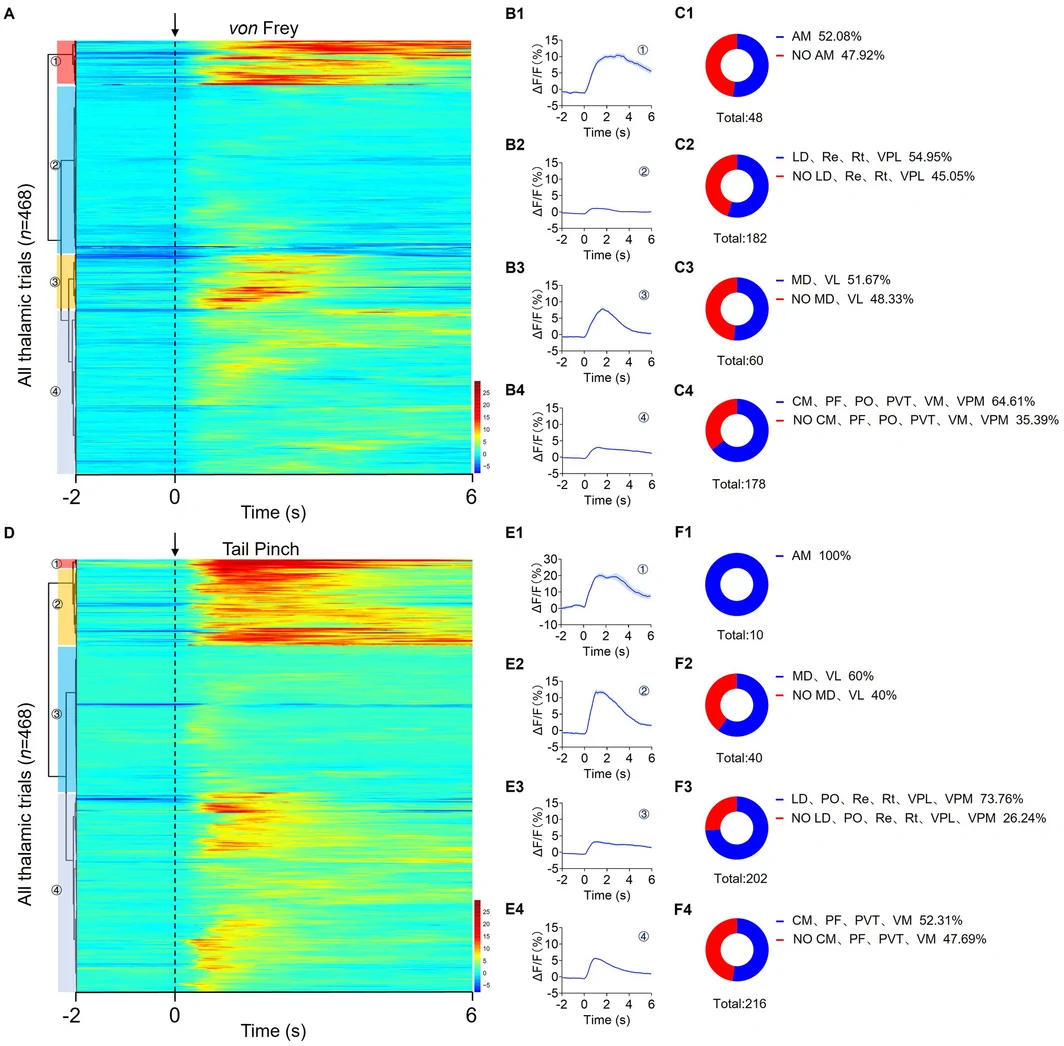
Hierarchical clustering analysis of Ca^2+^ signals evoked by *von* Frey and tail pinch stimuli across all trials in thalamic nuclei. (A) Hierarchical clustering analysis of Ca^2+^ signals evoked by *von* Frey stimulation across 468 trials from 13 thalamic nuclei. (B1–B4) Averaged Ca^2+^ signal traces for each identified cluster. (C1–C4) Proportions of trials contributed by different thalamic nuclei within each cluster. (D) Hierarchical clustering analysis of Ca^2+^ signals evoked by tail pinch stimulation across 468 trials from 13 thalamic nuclei. (E1–E4) Averaged Ca^2+^ signal traces for each cluster. (F1–F4) Proportions of trials contributed by different thalamic nuclei within each cluster.

Under *von* Frey filament stimulation (Figure 3A–C, Figures S2A and S3), Cluster 1 (ultra‐high signal) included only AM (52.08%); Cluster 2 (low signal) included LD, Re, Rt, and VPL (54.95%); Cluster 3 (high signal) included MD and VL (51.67%); and Cluster 4 (medium signal) included CM, PF, PO, PVT, VM, and VPM (64.61%).

Under tail pinch stimulation (Figure 3D–F, Figures S2B and S4), Cluster 1 (ultra‐high signal) included only AM (100%); Cluster 2 (high signal) included MD and VL (60%); Cluster 3 (low signal) included LD, PO, Re, Rt, VPL, and VPM (73.76%); and Cluster 4 (medium signal) included CM, PF, PVT, and VM (52.31%).

Combined analysis of mean Ca^2+^ signals and pooled trials under both noxious stimuli revealed a highly consistent hierarchical response pattern across the thalamus. AM consistently belonged to the ultra‐high signal cluster, MD and VL remained in the high‐signal cluster, medium‐signal regions (PF, CM, PVT, and VM) were primarily located medially, and low‐signal regions (VPL, VPM, PO, LD, and Rt) were mainly positioned laterally. These findings further support the existence of medial‐lateral distinctions in the thalamus during nociceptive processing.

### 3.4 Analysis of Thalamic Ca^2+^ Signals Evoked by Noxious Stimuli

**FIGURE 4:**
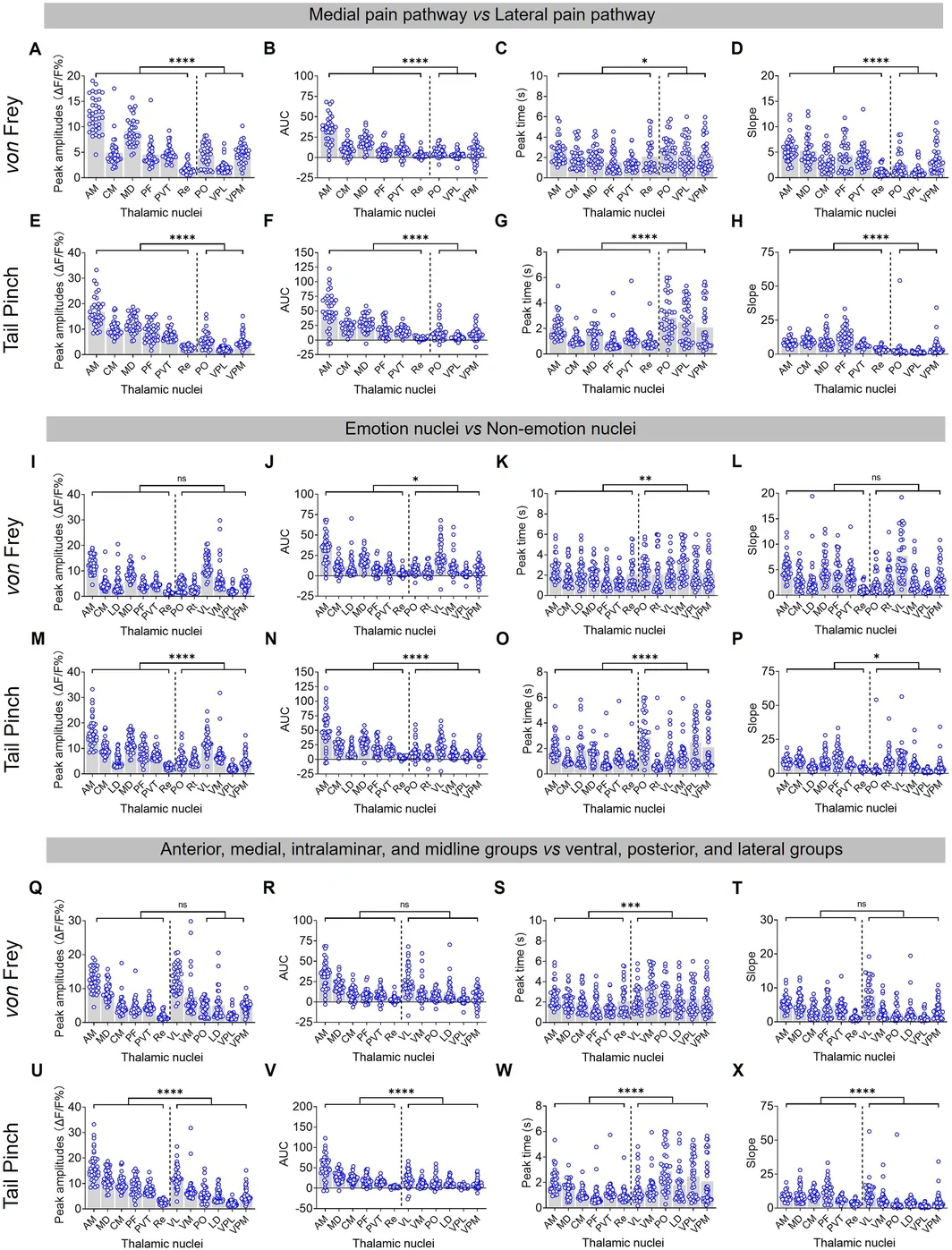
Comparison of Ca^2+^ signal responses to nociceptive stimulation across multiple thalamic nuclei. (A–D) Comparisons of Ca^2+^ signal AUC, peak amplitude, peak time, and slope evoked by *von* Frey stimulation between nuclei associated with the medial and lateral pain pathways. (E–H) Comparisons of Ca^2+^ signal AUC, peak amplitude, peak time, and slope evoked by tail pinch stimulation between nuclei involved in the medial and lateral pain pathways. (I–L) Comparisons of Ca^2+^ signal AUC, peak amplitude, peak time, and slope between emotion‐related and non‐emotion‐related nuclei in response to *von* Frey stimulation. (M–P) Comparisons of Ca^2+^ signal AUC, peak amplitude, peak time, and slope between emotion‐related and non‐emotion‐related nuclei in response to tail pinch stimulation. (Q–T) Comparisons of Ca^2+^ signal AUC, peak amplitude, peak time, and slope between medial and lateral thalamic groups in response to *von* Frey stimulation. (U–X) Comparisons of Ca^2+^ signal AUC, peak amplitude, peak time, and slope between medial and lateral thalamic groups in response to tail pinch stimulation. Unpaired *t*‐test, *p < 0.05, **p < 0.01, ***p < 0.001, ****p < 0.0001.

Previous observations indicated that thalamic responses to noxious stimuli exhibit a hierarchical pattern characterized by medio‐lateral anatomical distribution. To further validate this phenomenon, we analyzed and compared Ca^2+^ signals in thalamic nuclei involved in the medial and lateral pain pathways, based on classical nociceptive pathways. Under both stimulation conditions, we statistically analyzed the peak amplitude, AUC, peak time, and slope of Ca^2+^ signals in nuclei associated with the two pathways. The results showed that, regardless of *von* Frey filament or tail pinch stimulation, the medial pain pathway evoked Ca^2+^ signals with significantly higher peak amplitude, AUC, and slope, and earlier peak time compared to the lateral pathway (Figure 4A–H). These results suggest that the medial thalamic pain pathway exhibits greater excitability and responsiveness during noxious stimulus encoding.

Considering that pain perception includes emotional and cognitive components, we further categorized thalamic nuclei based on functional attributes into emotion‐related nuclei (AM, MD, CM, PF, PVT, Re, and LD) and non‐emotion‐related nuclei (VL, VM, VPM, VPL, PO, and Rt), and conducted the same comparisons (Figure 4I–P) [12, 41]. Under gentle *von* Frey filament stimulation, no significant differences in Ca^2+^ signals were observed between the two groups. However, under tail pinch stimulation, emotion‐related nuclei exhibited significantly higher peak amplitude, AUC, and slope, with earlier peak time compared to non‐emotion‐related nuclei. Previous studies reported that tail pinch typically causes minor tissue injury and elicits pronounced emotional responses, enhancing neural activity in emotion‐related thalamic nuclei [42, 43]. Our fiber photometry results corroborate these findings and further indicate that emotion‐related nuclei exhibit higher responsiveness to strong stimuli.

Based on classical anatomical subdivisions of the thalamus, we compared the medial and lateral thalamic groups (Figure 4Q–X). Under *von* Frey filament stimulation, no significant differences were observed between the two groups. However, under tail pinch stimulation, the medial group exhibited significantly higher peak amplitude, AUC, and slope, with earlier peak time compared to the lateral group. This further confirms the dominant role of medial thalamic regions in encoding strong noxious stimuli and aligns with the analyses based on pain pathways and functional attributes.

In summary, the thalamus exhibits pronounced medio‐lateral hierarchical response patterns during processing of noxious stimuli. Medial thalamic nuclei and emotion‐related nuclei display stronger and faster Ca^2+^ signal activation under intense stimulation, suggesting a key role in the emotional evaluation and higher‐order cognitive modulation of pain.

### 3.5 Analysis of Ca^2+^ Signals in Thalamic Nuclei Under Different Pain Intensities

**FIGURE 5:**
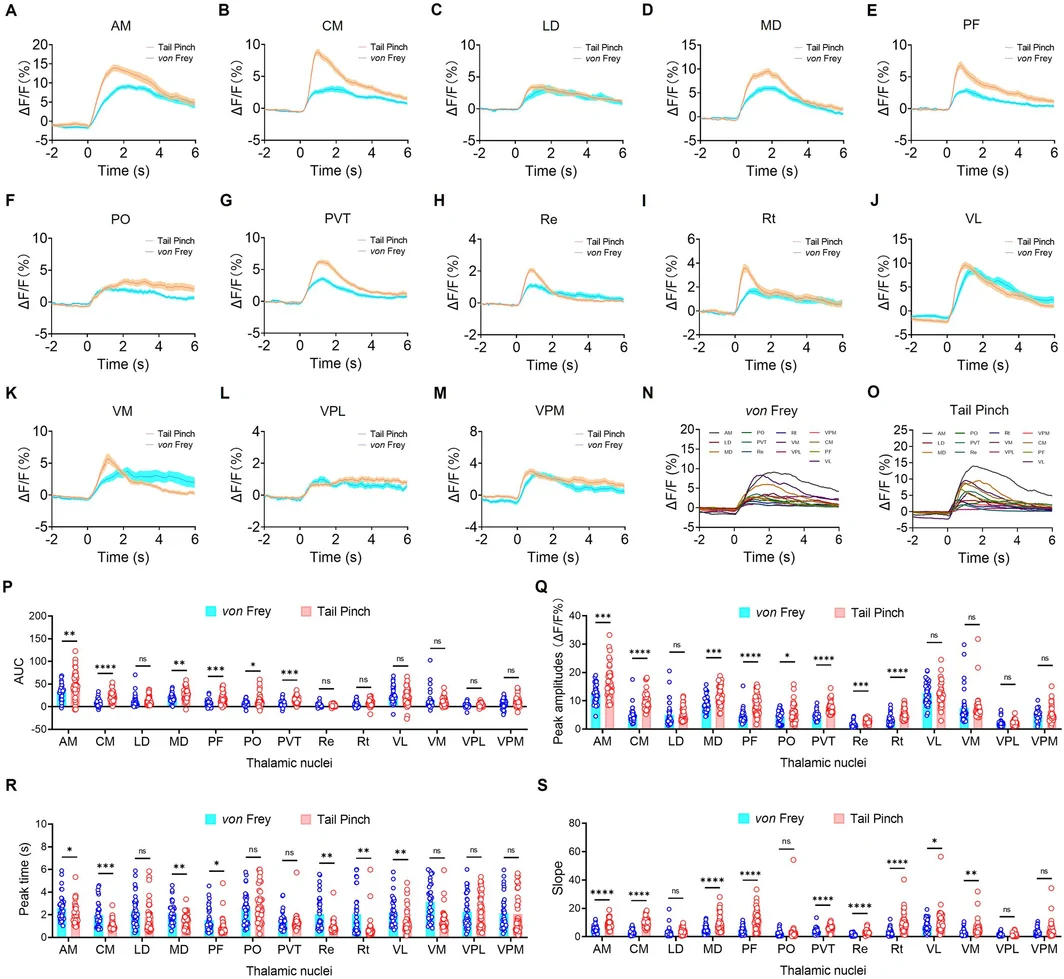
Comparison of Ca^2+^ signals in thalamic nuclei evoked by two different intensities of nociceptive stimulation. (A–M) Ca^2+^ signal traces of thalamic nuclei in response to *von* Frey filament and tail pinch stimulation. (N) Ca^2+^ signal traces of thalamic nuclei evoked by *von* Frey filament stimulation. (O) Ca^2+^ signal traces of thalamic nuclei evoked by tail pinch stimulation. (P) Comparison of Ca^2+^ signal AUC in thalamic nuclei evoked by *von* Frey and tail pinch stimulation. (Q) Comparison of Ca^2+^ signal peak amplitudes in thalamic nuclei evoked by *von* Frey and tail pinch stimulation. (R) Comparison of Ca^2+^ signal time‐to‐peak in thalamic nuclei evoked by *von* Frey and tail pinch stimulation. (S) Comparison of Ca^2+^ signal slopes in thalamic nuclei evoked by *von* Frey and tail pinch stimulation. Paired *t*‐test, *p < 0.05, **p < 0.01, ***p < 0.001, ****p < 0.0001.

Previous results showed that all 13 thalamic nuclei exhibited significant increases in Ca^2+^ signals following noxious stimulation, but the same nucleus exhibited differential Ca^2+^ responses to stimuli of varying intensities in the present study (Figure 5A–O). Prior studies have reported that the MD, PF, and PVT nuclei exhibit intensity‐dependent neural activity in response to noxious or stress stimuli of varying intensities [11, 44]. To quantify these differences, we performed paired *t*‐tests on the Ca^2+^ signal AUC, peak amplitude, peak time, and slope for each thalamic nucleus under *von* Frey filament and tail pinch stimulation (Figure 5P–S). The AUC analysis revealed that AM, CM, LD, MD, PF, PO, and PVT exhibited significant differences in response to different pain intensities, whereas Re, Rt, VL, VM, VPL, and VPM did not show significant AUC differences between stimuli. Analysis of peak amplitude showed that AM, CM, MD, PF, PO, PVT, Re, and Rt exhibited significant differences in peak responses across pain intensities, whereas LD, VL, VM, VPL, and VPM did not. Nuclei showing significant differences, particularly those differing in both AUC and peak amplitude (AM, CM, MD, PF, PO, and PVT), may contribute to encoding pain intensity. Additionally, we analyzed peak time and slope of Ca^2+^ signals across nuclei under different stimulus intensities. Compared with *von* Frey stimulation, AM, CM, MD, PF, Re, Rt, and VL exhibited shorter peak times under tail pinch stimulation, whereas LD, PO, VM, VPL, and VPM showed no differences in peak times between the two stimulus intensities. Similarly, the slope of Ca^2+^ signals was steeper for AM, CM, MD, PF, PVT, Re, Rt, VL, and VM under tail pinch compared to *von* Frey stimulation, whereas LD, PO, VPL, and VPM showed no significant differences in slope between the two intensities.

In summary, thalamic nuclei exhibit marked differences in Ca^2+^ responses to varying pain intensities. AM, CM, MD, and PF showed higher AUC and peak amplitude, shorter peak times, and steeper rise slopes under high‐intensity stimulation, suggesting that these regions may play key roles in encoding pain intensity, as well as in emotional processing and hierarchical pain assessment. In contrast, LD, VL, VM, VPL, and VPM displayed similar Ca^2+^ responses under both stimulus intensities, indicating that these nuclei may primarily convey stable sensory input and are less sensitive to variations in stimulus intensity.

### 3.6 Projection Differences Between PF and PO

In the previous analysis, we compared the Ca^2+^ signal characteristics of 13 thalamic nuclei in response to noxious stimuli. The results showed that within the dorsal thalamus, PF exhibited the shortest peak time, whereas PO showed the longest, indicating a significant temporal difference between the two nuclei (Figure S5A,B). To further investigate whether this temporal difference is associated with projection patterns, we conducted single neuron morphological analyses of PF and PO output projections.

**FIGURE 6:**
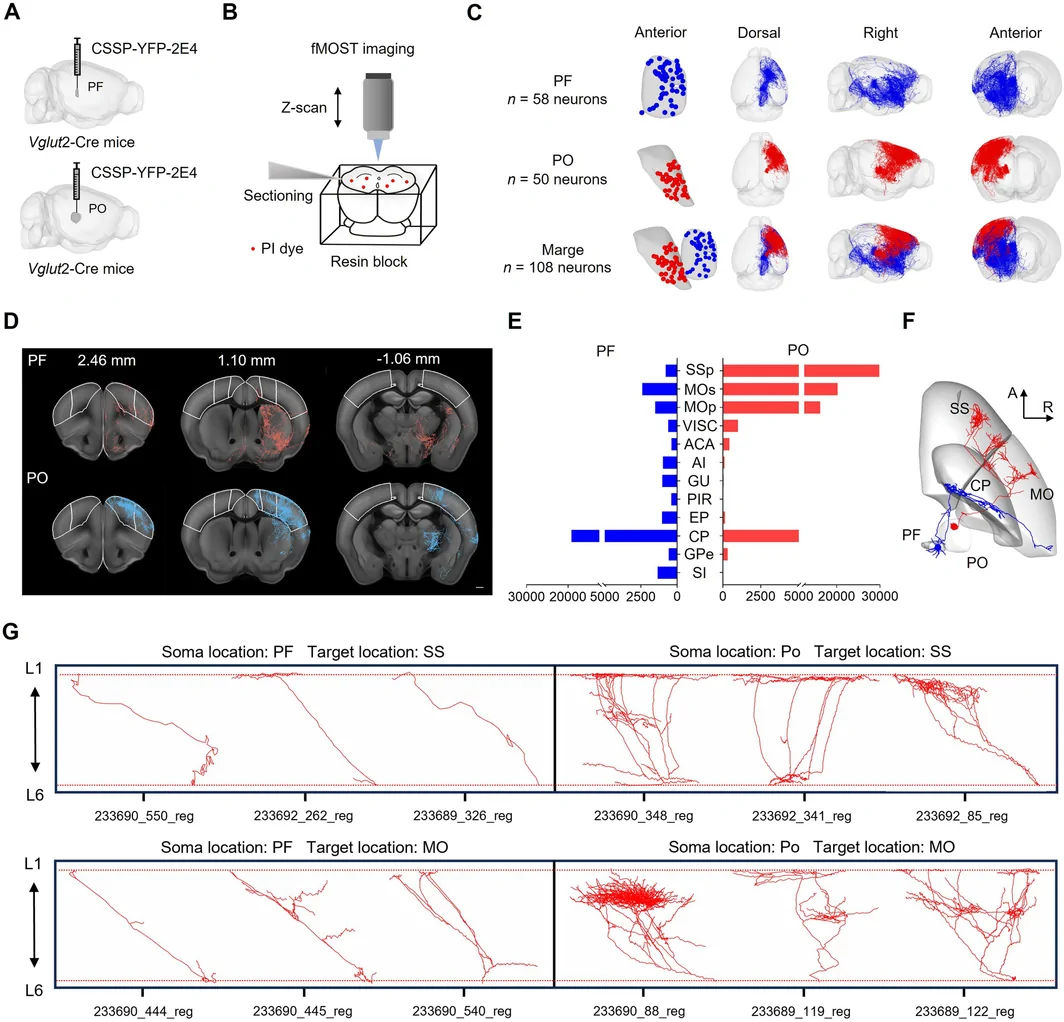
Projection patterns of Vglut2 neurons in the PF and PO. (A) Schematic illustration of the viral injection strategy. (B) Schematic diagram of *HD*‐*f*MOST imaging. (C) Visualization of the somatic distribution and projection patterns of Vglut2 neurons in the PF and PO from multiple viewing angles. (D) Coronal‐section visualization of projection patterns of Vglut2 neurons in the PF and PO. (E) Quantitative analysis of projection strength from the PF and PO. (F) Representative projection patterns of single Vglut2 neurons in the PF and PO. (G) Laminar distribution of Vglut2 neuronal projections from the PF and PO within sensory and motor cortices.

We used animals from Groups 15 and 16 (Vglut2‐Cre mice) and injected CSSP‐YFP‐2E4 virus into PF and PO according to grouping, sparsely labeling glutamatergic neurons (Figure 6A). Subsequently, *HD*‐*f*MOST combined with a semi‐automated tracing algorithm was used to reconstruct the dendritic and axonal structures of the labeled neurons (Figure 6B,C). A total of 108 neurons from 10 mice were reconstructed, including 58 PF neurons and 50 PO neurons.

Analysis of whole‐brain axonal distribution revealed distinct projection patterns for PF and PO neurons across coronal sections (Figure 6D). We then compared the top 30 brain regions receiving the strongest projections from PF and PO and found that they shared 12 common target areas. Quantitative analysis showed that PF neurons primarily projected to the caudoputamen (CP), whereas PO neurons mainly projected to cortical regions, including the primary somatosensory cortex (SSp), primary motor cortex (MOp), and secondary motor cortex (MOs) (Figure 6E, Figure S5C,D). Additionally, representative single‐neuron projection maps of PF and PO were presented to visually illustrate differences in their output patterns (Figure 6F).

Detailed analysis of thalamocortical projections revealed distinct axonal distribution patterns in the cortex for PF and PO (Figure 6G). PF axons displayed sparse, point‐to‐point distributions with limited coverage per axon, whereas PO axons formed denser, clustered patterns that spanned multiple cortical layers.

In summary, PF and PO differ not only in the temporal characteristics of Ca^2+^ signals induced by noxious stimuli but also in their downstream projection targets and cortical axonal distributions. These structural features provide important morphological evidence that PF and PO may play distinct functional roles in pain information processing.

## 4 Discussion

### 4.1 The Thalamus Is Not a Passive Relay but Composed of Functionally Specialized Nuclei

Traditionally, the thalamus has been considered primarily as a relay for ascending sensory information. However, accumulating evidence indicates that it is not a structurally or functionally uniform relay, but rather consists of multiple highly specialized nuclei, each contributing to multidimensional processing, including sensory discrimination, attention‐arousal, and emotion‐cognition functions [8, 9, 10]. In the present study, we observed that all 13 thalamic nuclei exhibited significant Ca^2+^ signal activation in response to noxious somatic stimulation, consistent with previous fMRI and c‐FOS immunohistochemical studies, further supporting the notion that the thalamus is globally activated during nociceptive input [13, 20].

Notably, our results further revealed that the thalamic response to noxious stimuli is not uniform but exhibits a pronounced medial‐lateral gradient. Specifically, Ca^2+^ signal intensity was higher in medial pain pathways than in lateral pathways, greater in emotion‐related nuclei compared to non‐emotion‐related nuclei, and stronger in medial thalamic groups than in lateral groups. This spatial distribution is consistent with the “medial‐lateral pain pathway” theory established in humans and rodents, in which medial nuclei preferentially process the affective‐motivational dimensions of pain, whereas lateral nuclei primarily transmit sensory‐discriminative information [13]. Furthermore, a recent human electroencephalogram (EEG) study reported that, compared with ventral nuclei, the medial and intralaminar nuclei exhibit earlier and stronger event‐related potentials (ERPs) responses under conditions involving awareness, emotion, and noxious stimulation [17]. Despite differences in recording methods, these findings consistently support the heightened sensitivity of medial thalamic nuclei in nociceptive processing.

Why are medial nuclei more sensitive to noxious stimuli? We hypothesize that this phenomenon arises from multiple converging factors. First, medial nuclei may be more susceptible to ascending nociceptive drive and cortical feedback. Strong noxious stimuli could markedly enhance inputs through the spinal cord‐brainstem‐thalamus pathway, particularly projecting to medial, midline, and intralaminar nuclei, thereby providing these nuclei with stronger nociceptive input than lateral nuclei. Second, the affective‐motivational pain circuits formed by medial thalamic nuclei with the ACC, PFC, and IC may further amplify cortical feedback under strong stimulation, enhancing medial nucleus excitability. Multiple studies indicate that medial nuclei connections with the prefrontal‐limbic system are crucial for the emotional‐motivational dimensions of pain and can be strongly driven by ascending nociceptive inputs [20, 45, 46]. Third, Rt may impose rhythmic inhibitory control on lateral thalamic nuclei, limiting their excitability. As an inhibitory structure enveloping the thalamus, Rt exerts critical gating and selective modulation during sensory transmission. Previous studies have shown that Rt plays a key role in sensory gating, and activation of the Rt‐ventrobasal thalamic (VB) pathway can attenuate the transmission of pain and itch signals [47, 48]. Additionally, differences in neurotransmitters, receptor distribution, and local microcircuits among thalamic nuclei may result in distinct synaptic gain and excitability [49]. In summary, the medial thalamic nuclei exhibit more pronounced responsiveness to noxious stimuli, suggesting that they play a critical role in pain‐related emotional processing and the regulation of defensive behaviors.

### 4.2 Differential Response Patterns of Individual Thalamic Nuclei to Stimuli of Varying Intensities

When comparing the AUC and peak amplitude of Ca^2+^ signals evoked by nociceptive stimuli of different intensities within the same thalamic nuclei, we observed that AM, CM, MD, PF, PO, and PVT exhibited intensity‐dependent Ca^2+^ responses. Notably, all of these nuclei are functionally associated with affective processing. In contrast, the AUC and peak amplitude of Ca^2+^ signals in VL, VM, VPL, and VPM did not show significant differences across stimulus intensities. We speculate that these differences are closely related to the intrinsic functional properties of the respective nuclei. VL serves as a critical hub for motor information integration, receiving major inputs from the cerebellum and globus pallidus and projecting to the motor cortex, thereby playing a pivotal role in motor coordination, postural control, and movement execution [50, 51]. In the present study, both types of stimuli elicited motor responses in the animals, which may account for the lack of significant differences in Ca^2+^ signals between the two conditions. Previous studies have shown that the VM primarily participates in maintaining cortical excitability and motor preparation rather than classical sensory processing, which may explain its insensitivity to stimulus intensity [52, 53]. VPL and VPM are primarily responsible for processing primary somatosensory information, with VPL relaying sensory inputs from the trunk and limbs, and VPM processing sensory inputs from the face and head [32, 54]. Interestingly, despite the hindpaw and tail stimulation paradigms used in the present study, the responses observed in the VPL were not stronger than those in the VPM. Given that fiber photometry measures population‐level Ca^2+^ activity rather than direct afferent input strength, the recorded signals may reflect the combined influence of sensory inputs, local circuit integration, neuronal composition, intrinsic cellular properties, and network‐level interactions. Therefore, the mechanisms underlying this observation are likely multifactorial and remain to be further elucidated. In addition, because VPM primarily receives sensory inputs from the orofacial region, differences in stimulation sites may also contribute to the observed variability in its responses. Collectively, these findings indicate that distinct thalamic nuclei exhibit functionally specific patterns of responsiveness to pain intensity, consistent with their specialized roles in sensory and affective processing.

### 4.3 Temporal Characteristics of Ca^2+^ Signals Evoked by Noxious Stimuli Across Distinct Thalamic Nuclei

The temporal dynamics of Ca^2+^ signals are often overlooked in neuroscience research, despite their substantial physiological significance. Previous work demonstrated that in chloroquine‐induced non‐histaminergic itch, Ca^2+^ signals in certain brain regions exhibit transient, pulse‐like increases followed by rapid decay, whereas histamine‐dependent itch elicits distinctly different Ca^2+^ dynamics [23]. Consistent with these findings, we observed comparable temporal patterns in our experiments. Within the dorsal thalamus, PF exhibited the shortest Ca^2+^ peak time, whereas PO showed the longest, indicating a significant temporal divergence between these nuclei.

Previous studies have shown that nociceptive information is primarily conveyed to the VPL and VPM via the spinothalamic tract and subsequently transmitted to the cerebral cortex, where corticothalamic feedback modulates thalamic activity. More recent work has suggested that the genuine thalamic response to nociceptive stimulation is primarily reflected in the early negativity (EN) component of event‐related potentials, rather than the conventional N1 component, suggesting a multistage regulatory role of the thalamus in pain processing [54]. Accordingly, the Ca^2+^ signals recorded in the thalamus likely represent the integrated outcome of multiple converging and diverging pathways. Accordingly, Ca^2+^ signals recorded in the thalamus likely represent the integrated outcome of multiple converging afferent inputs and efferent outputs.

Against this background, we performed single‐neuron‐level morphological analyses of the output projections from PF and PO. The results revealed marked differences in downstream projection targets and intracortical distribution patterns between the two nuclei. Specifically, Vglut2 neurons in the PF predominantly projected to striatal‐related regions, exhibiting sparse and point‐to‐point axonal innervation within the cortex, whereas Vglut2 neurons in the PO primarily projected to sensory and motor cortices, forming dense, clustered axonal arborizations spanning multiple cortical layers. Consistent with these findings, previous functional studies have demonstrated a division of labor between PF and PO in pain processing, whereby the PO‐S1 pathway predominantly mediates tissue injury‐induced hyperalgesia, whereas the PF‐ACC pathway preferentially contributes to hyperalgesia under depression‐like states [29]. This work suggests that distinct thalamic nuclei may selectively drive sensory and affective components of pain through their specific cortical projections under different pathological conditions. This notion is also consistent with our observation of medial‐lateral differences in pain‐evoked Ca^2+^ signals, wherein medial thalamic nuclei preferentially engage in affective processing, while lateral nuclei are biased toward sensory discrimination.

Considering the distinct differences in Ca^2+^ peak time between PF and PO, we speculate that these projection patterns may be closely associated with their distinct temporal profiles and functional roles. The downstream axonal projections of PF are relatively sparse and exhibit a point‐to‐point distribution, suggesting that this nucleus may be rapidly recruited during the early phase of stimulation and modulate pain‐related affective states and behavioral responses via basal ganglia‐thalamic and related circuits. By contrast, the axons of PO form dense, clustered arborizations within sensory and motor cortices, spanning multiple cortical layers. This high‐density, multilayer‐spanning architecture is well suited to support sustained information integration and cortical encoding, which likely accounts for the delayed Ca^2+^ peak observed in PO. Together, these results indicate that functional specialization among thalamic nuclei in response to nociceptive stimulation is reflected in both their projection architecture and temporal response dynamics, providing a structural basis for hierarchical pain processing.

Nevertheless, several limitations should be acknowledged. First, the use of a pan‐neuronal Ca^2+^ indicator precluded discrimination between responses of distinct neuronal subtypes. In addition, while the present study examined the downstream projection patterns of PF and PO, differences in their upstream inputs may also influence the temporal dynamics of the observed Ca^2+^ responses. Moreover, differences in viral expression efficiency, neuronal density, cellular composition, and neurotransmitter profiles across thalamic nuclei may influence the magnitude of the recorded Ca^2+^ signals. Furthermore, although nociceptive stimulation was used throughout this study, the recorded Ca^2+^ signals may not exclusively reflect nociceptive processing, as such stimuli can also engage movement‐related, attention‐related, arousal‐related, and defensive behavioral processes. Second, bilateral stimulation was not performed, limiting assessment of potential lateralization effects within the thalamus. Finally, this study focused exclusively on mechanical nociceptive stimuli and did not examine thalamic responses to thermal or inflammatory pain modalities.

In summary, using fiber photometry, this study characterized Ca^2+^ signaling dynamics across multiple thalamic nuclei under different intensities of nociceptive stimulation and revealed their distinct response patterns to nociceptive stimulation. These findings provide new experimental evidence for functional segregation and encoding mechanisms within the thalamus during pain processing and lay a foundation for future investigations into pain‐related neural circuits and potential therapeutic targets.

## 5 Conclusion

In summary, nociceptive stimulation elicited rapid increases in Ca^2+^ signals across all recorded thalamic nuclei in this study, including AM, CM, LD, MD, PF, PO, PVT, Re, Rt, VL, VM, VPL, and VPM; however, significant differences were observed among nuclei in the AUC, peak amplitude, peak time, and slope of the evoked Ca^2+^ signals. Cluster analysis further revealed a clear spatial pattern, with medially located thalamic nuclei generally exhibiting stronger Ca^2+^ responses, whereas laterally located nuclei displayed relatively weaker responses. In addition, thalamic nuclei differed in their sensitivity to stimulus intensity, with some showing pronounced intensity‐dependent Ca^2+^ responses, whereas others exhibited minimal changes. Collectively, these findings provide new experimental evidence supporting the view that the thalamus acts as a distributed and hierarchical hub for pain information processing.

## Author Contributions

Y.‐Q.L., F.L., and Z.‐P.C., designed the study, provided the funding support, and edited the manuscript; Y.‐H.L. wrote the manuscript and acquired the data; F.L., and T.‐Y.Z. provided guidance for *f*MOST imaging; F.‐S.H., J.C., L.‐M.L., S.‐S.T., Y.‐J.C., and J.‐Q.Z. completed the data analyzed; All authors read and approved the final vision of the manuscript.

## Funding

This study was supported by the Brain Science and Brain‐like Intelligence Technology—National Science and Technology Major Project (No. 2021ZD0204403 to Y‐Q. Li) and National Natural Science Foundation of China (Nos. 82130034, 82471254, and 82221001 to Y‐Q. Li).

## Ethics Statement

All animal procedures performed in this study were conducted in strict compliance with the international standards of Animal Care Guidelines and have been approved by the Institutional Animal Ethics Committee of Air Force Medical University (ID: SYXK (Shannxi) 2024‐003).

## Conflicts of Interest

The authors declare no conflicts of interest.

## Supporting information


**Figure S1:** Injection sites in 13 thalamic nuclei and their corresponding Nissl staining.
**Figure S2:** Distribution of the 13 thalamic nuclei across clusters under two types of nociceptive stimulation.
**Figure S3:** Detailed clustering analysis data of all trials under *von* Frey stimulation.
**Figure S4:** Detailed clustering analysis data of all trials under Tail Pinch stimulation.
**Figure S5:** Quantitative comparison between PF and PO.

## Data Availability

The data that support the findings of this study are available from the corresponding author upon reasonable request.
