# Supplementary material for: Hierarchically and Temporally Distinct Ca2+ Signaling Across Thalamic Nuclei During Nociceptive Stimulation
Source: CNS Neurosci Ther. 2026 Aug 31;32(9):e71119. doi: 10.1002/cns.71119 (PMC13531012; doi:10.1002/cns.71119)
Supplement: Supplementary file 1 [file CNS-32-e71119-s001.docx]

Figure S1. Injection sites in 13 thalamic nuclei and their corresponding Nissl staining.

(A1-M1) Injection sites of the 13 thalamic nuclei. (A2-M2) Corresponding Nissl-stained sections of the injection sites in the 13 thalamic nuclei. AVVL, anteroventral thalamic nucleus, ventrolateral part; VA, ventral anterior thalamic nucleus; AV, anteroventral thalamic nucleus; IMD, intermediodorsal thalamic nucleus; fr, fasciculus retroflexus; LPMR, lateral posterior thalamic nucleus, mediorostral part; LPLR, lateral posterior thalamic nucleus, laterorostral part; DV3, dorsal third ventricle; PT, parataenial thalamic nucleus; iC, internal capsule; VPPC, ventral posteromedial thalamic nucleus, parvicellular part; ZID, zona incerta, dorsal part. Scale bar = 200 μm.

Figure S2. Distribution of the 13 thalamic nuclei across clusters under two types of nociceptive stimulation.

Figure S3. Detailed clustering analysis data of all trials under *von* Frey stimulation.

Figure S4. Detailed clustering analysis data of all trials under Tail Pinch stimulation.

Figure S5. Quantitative comparison between PF and PO.

(A) Hierarchical clustering analysis of the average Ca²⁺ signals evoked by tail pinch across 13 thalamic nuclei; black dots indicate peak responses. (B) Peak time of Ca²⁺ signals evoked by tail pinch across 13 thalamic nuclei. (C) Quantification of projection strength of Vglut2 neurons in the PF. (D) Quantification of projection strength of Vglut2 neurons in the PO.
